# Balancing Costs and Clinical Outcomes: A Cost-Effectiveness Study of PICC Types Across Chinese Healthcare Settings

**DOI:** 10.3390/jmahp13040049

**Published:** 2025-09-29

**Authors:** Jia Xu, Hossein Zare, Xia Yan, Herng-Chia Chiu, Renan Castillo

**Affiliations:** 1 Department of Health Policy and Management, Johns Hopkins Bloomberg School of Public Health, Baltimore, MD 21202, USA; hzare1@jhu.edu (H.Z.); rcastil1@jhu.edu (R.C.); 2 Perking University People’s Hospital, Beijing 100140, China; yanxia3910@sina.com; 3 Institute for Hospital Management, Tsinghua Shenzhen International Graduate School, Shenzhen 518055, China

**Keywords:** peripherally inserted central catheter, cost-effectiveness, quality-adjusted life year, incremental cost-effectiveness ratio, healthcare economics

## Abstract

Long-term intravenous therapies often necessitate the use of peripherally inserted central catheters (PICCs). Antimicrobial-coated PICCs have been introduced to minimize central line-associated bloodstream infections (CLABSIs). A decision-analytic cost-effectiveness model was developed from a societal perspective, utilizing real-world data concerning PICC-related complications and costs from Class 3A hospitals and community hospital settings in China. The analysis compared the quality-adjusted life years (QALYs) for patients receiving antimicrobial-coated PICCs versus standard PICCs, with catheter-associated costs included. Incremental cost-effectiveness ratios (ICERs) were calculated in Chinese Yuan (CNY) per QALY gained. Patients with antimicrobial-coated PICCs experienced slightly fewer complication-related events, leading to significantly lower costs for managing complications. In the Class 3A hospital setting, the average total cost per patient was lower with antimicrobial-coated PICCs (CNY 62,800) compared to standard PICCs (CNY 102,900), primarily due to the reduced expenses for treating CLABSIs and related unknown fever. The ICER demonstrated that the coated PICC was the strongest option, showing a negative ICER (cost-saving of approximately CNY 4 million per QALY gained in the base-case Class 3A hospital scenario). In community hospital care scenarios, the cost advantage of antimicrobial-coated PICCs remained. Thus, the antimicrobial-coated PICC strategy was cost-saving, providing equal or improved health outcomes at lower costs in China’s medical center and community hospitals, making it a more efficient choice for long-term vascular access.

## 1. Introduction

Central line-associated bloodstream infection (CLABSI) is a major concern associated with central venous catheters (CVCs). Compared to CVCs, peripherally inserted central catheters (PICCs) have a lower infection rate and are easier to manage, making them indispensable in modern healthcare for cancer patients and others needing long-term venous infusion [1,2]. PICCs are widely used for prolonged intravenous therapies, such as chemotherapy or antibiotic therapy. Various types of PICC are available, and understanding their cost-effectiveness is crucial for making informed healthcare decisions. Two distinct types have garnered attention for their potential to reduce catheter-related complications: Antimicrobial-coated PICCs and standard PICCs.

The advent of antimicrobial coating represents a significant advancement in CLABSI prevention [3,4]. A randomized controlled trial comparing antimicrobial-impregnated CVC versus standard CVC showed that CLABSI risk reduction (RR) with chlorhexidine/silver sulfadiazine-coated CVC was 0.73 (95% confidence interval [CI]: 0.57–0.94) [5]. However, few randomized controlled trials (RCTs) have focused on PICCs. One RCT comparing miconazole- and rifampicin-impregnated PICCs with standard PICC found no significant difference in clinical outcomes. Another RCT comparing antimicrobial-coated PICCs with standard PICCs reported no significant difference in reducing CLABSI risk (*p* = 0.61) or reducing VTE events (*p* > 0.99) [6,7].

Few studies have examined the economic sustainability of different types of PICCs. The distinctive costs associated with nursing care and complication management make assessing the economic impact of PICC use in long-term intravenous therapy particularly challenging. To address this gap, we conducted a cost-effectiveness analysis (CEA) utilizing secondary data to evaluate a novel catheter design and its associated complications. In China, community hospitals generally incur lower charge expenses compared to Class 3 hospitals, due largely to government policies aimed at encouraging the utilization of community healthcare resources for routine medical needs. Studies on China’s healthcare system reforms suggest that expanding primary and community care capacities effectively addresses the imbalance between escalating healthcare demands and limited resources at tertiary hospitals [8,9,10,11].

While incorporating these societal insights, this study evaluates the economic implications of antimicrobial-coated PICCs relative to standard PICCs, specifically comparing hospital-based care with community-based catheter management.

## 2. Materials and Methods

### 2.1. Study Design and Sample Population

This cost-effectiveness analysis (CEA) was based on data from Peking University People’s Hospital, a Class 3A hospital in China. All patients enrolled in the analysis underwent PICC procedures in the hematology departments from September 2020 to May 2023. As of 31 May 2023, Peking University People’s Hospital had a total of 430 beds, and during this period, 6923 PICC units were inserted.

The economic evaluation was based on data from adult hematology patients who required either a bone marrow transplant or chemotherapy. The analysis included two different types of catheters: antimicrobial-coated PICCs and standard PICCs. A total of 224 patients were included in the analysis.

Cost data for treatment and PICC-related devices were obtained from both the Class 3A hospital and a representative community hospital located in central Beijing.

### 2.2. Evaluation Points

The intravenous access procedures adhered to the CLABSI prevention program, which included the CDC’s recommended bundle strategies: maximum sterilized barrier, antibacterial-based antiseptic skin preparation, hand hygiene, and related training [12,13,14,15,16]. The 3 PICC insertion nurses were a dedicated team accredited and certified by the Chinese Nurse Society. Each nurse had performed at least 50 procedures per year to maintain consistent, high-quality insertion techniques. The observation period was 90 days, with regular follow-ups to maintain catheter function. The standard follow-up procedures included insertion point care, ultrasonographic assessment of CVC-related thrombosis, and catheter function checks. The main evaluation points of PICCs were the first attempted insertion success rate; catheter-related complications, which included the catheter-related bloodstream infections; thrombosis; and other catheter-related local complications. The CLABSI was determined according to the laboratory-confirmed bloodstream infection criteria during the PICC indwelling time, excluding mucosal barrier injury laboratory-confirmed bloodstream infection (MBI-LCBI) [16,17,18]. Fever of unknown origin among patients was recorded according to patient-reported symptoms and clinical assessments that did not meet the CLABSI criteria. Quality-adjusted life-years (QALYs) were not gathered directly, as referred to in other reported articles, and the direct costs related to PICC insertion and maintenance were obtained from the medical record [19,20,21,22,23].

### 2.3. Cost Evaluation Measures

The cost analysis was conducted from a societal perspective, reflecting public hospital expenses reimbursed under China’s Urban and Rural Residents Basic Medical Insurance (URRBMI). We calculated the following in both medical center and community hospital settings: the price of the standard PICC, estimated price of the antimicrobial-coated PICC (CNY/piece) (This PICC was not available in China in 2020), average costs of insertion and maintenance once per patient (CNY/procedure), average costs for CLABSI diagnosis and treatment (CNY/episode), and average duration of hospitalization per therapy stage (days). Indirect disease-related costs, such as productivity loss, were not included, as they were considered similar across both groups. However, costs associated with complications and unplanned catheter withdrawal—such as reinsertion and symptomatic treatment—were included.

### 2.4. Currency Rate and Conversion

All costs were calculated according to real average prices reported from 2019 to 2023 in the Beijing Municipal Price Bureau. All costs are presented in Chinese Yuan (CNY). The average official currency conversion rate of the China Central Bank from January 2020 to December 2023 was CNY 1 equal to USD 0.1475.

### 2.5. Cost-Effectiveness Analysis

A decision tree model was developed to compare two long-term infusion strategies: the antimicrobial-coated PICC and the standard PICC. The model incorporated discrete outcome probabilities related to device removal, maintenance, and infectious and non-infectious complications during the treatment episode. This structure enables a clear depiction of potential clinical pathways and corresponding economic outcomes.

The primary outcome was the incremental cost-effectiveness ratio (ICER), calculated as the cost per QALY gained. Cost-effectiveness analysis (CEA) was chosen as the evaluation method because it allows for a comprehensive comparison of both the clinical benefits and economic impact of each option.

### 2.6. Sample Size Calculation

This retrospective cohort study included adult patients who either received their first PICC insertion or underwent a new insertion at least 12 months after a prior one, with an expected survival time of over one year. We aimed to compare the incidence of central line-associated bloodstream infection (CLABSI) between antimicrobial-coated and standard PICCs. Based on prior literature, CLABSI rates were reported as 0.2% versus 5.3%, respectively. A total of 224 patients were included, which meets the estimated requirement for 80% power under a superiority design, assuming a one-sided α of 0.025.

### 2.7. Statistical Analysis

Data were analyzed using Stata/SE 15.1 and TreeAge Pro Healthcare 2023 (version R1.2), specialized software for health outcome modeling. Continuous variables are reported as means with standard deviations (SDs), while categorical variables are summarized using counts and percentages. Data for expenditure calculations of complication treatments were collected using the micro-costing method alongside standard patient care throughout the study [24,25,26]. The mean daily care expenditure for each scenario was used to estimate the average cost of each procedure associated with central vascular access [27].

## 3. Results

In our analysis, a total of 224 patients (136 male and 88 female) with hematologic diseases, aged 18–83 years (mean age, 42.34), required PICCs. The mean indwelling time of the antimicrobial-coated PICCs was 62.81 (SD 27.98) days, while the standard PICCs had an indwelling time of 69.04 (SD 26.65) days (*p* = 0.089). The baseline characteristics of all patients included in the study are presented in Table 1.

A total of 224 patients were included in this study, with four experiencing failure on the first insertion attempt. The remaining 220 patients were followed for the entire catheter indwelling period, during which CLABSI, fever of unknown origin, and local complications were documented. Eight patients met the criteria for bloodstream infection (BSI): three were diagnosed with CLABSI and five with MBI-LCBI. Although 36 patients developed CLABSI-related symptoms, none met all the diagnostic criteria, and other sources of infection were excluded. In the standard PICC group, three patients developed CLABSI; however, the difference between the two groups was not statistically significant (*p* = 0.076). Fever of unknown origin occurred in 17 patients (15.18%) with antimicrobial-coated PICCs and 20 patients (19.05%) with standard PICCs (*p* = 0.449) (Table 1). The unplanned removal of the catheter in the antimicrobial-coated PICC group was 31 (30.39%), while in the standard PICC group, it was 26 (25.24%, *p* = 0.411).

### 3.1. Cost Analysis

The Beijing Municipal Price Bureau for Class 3A hospitals provided the prices of the standard PICC, nursing expenses per patient, and laboratory test fees. Until 2023, there was no price label for the Antimicrobial-Coated PICC. To complete the decision tree model, the estimated price of the antimicrobial-coated PICC was set to CNY 2300, based on the price of a standard PICC (CNY 2100).

China’s Basic Medical Insurance covers regular care and PICC insertion costs for Urban and Rural Residents. The direct costs of PICC are listed in Table 2, calculated based on the average per-care costs, including costs per insertion and maintenance in 2020. Diagnosing CLABSI requires at least two central catheter blood cultures per lumen and two peripheral blood cultures, as per hospital protocol. The average cost for diagnosing CLABSI was CNY 1332.77 per event, while the average treatment cost per patient for each CLABSI event was CNY 87,147.08, which included parenteral antibiotics, catheter removal, and other empirical therapies.

The expenses charged by community hospitals are lower than those of Class 3 hospitals. Routine catheter maintenance and care can be managed at community hospitals. During the COVID-19 period, decentralized follow-up became a common practice, and 12% of patients received catheter maintenance in community hospitals. Therefore, this study estimated all follow-up period costs for catheter maintenance based on the community hospital charge list.

### 3.2. Decision Tree Model

**Figure 1:**
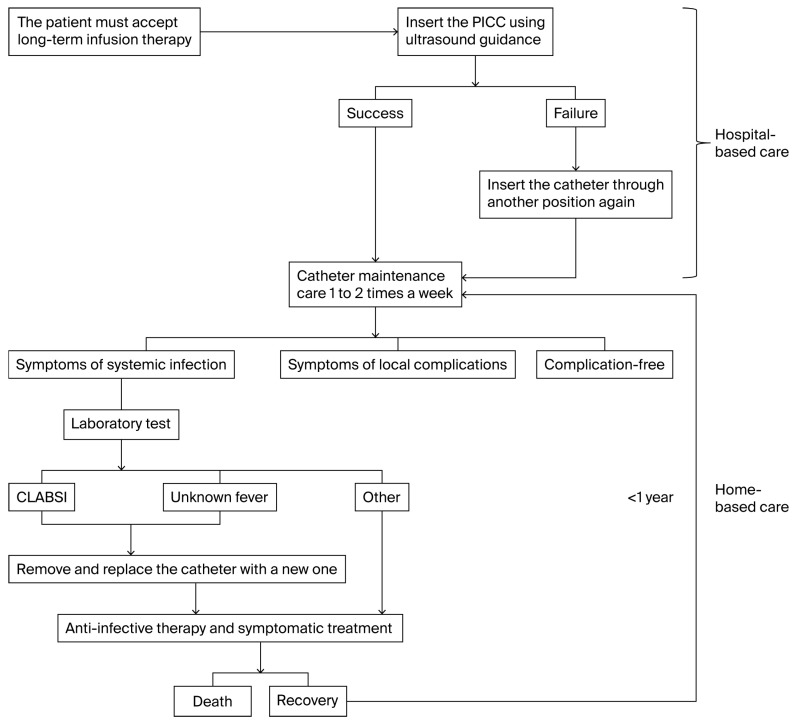
Decision Tree Model structure from PICC insertion to maintenance.

The decision tree for economic analysis illustrates the trajectory of the two types of PICCs and the two different healthcare settings (Figure 1). The rollout of the decision tree indicates that the antimicrobial-coated PICC is a more advanced device for managing expenses related to catheter-related complications in community hospital settings, with a total expense of CNY 61,235.43 for 90 days of antimicrobial-coated PICC in a community hospital setting.

### 3.3. Cost-Effectiveness Analysis

Cost-effectiveness analysis (CEA) quantifies the cost per unit of clinical effect gained [29,30,31,32]. Since the cost of standard PICCs was lower than that of antimicrobial-coated PICCs, we calculated the incremental cost-effectiveness ratio (ICER) to evaluate their comparative economic value.

#### 3.3.1. Cost-Effectiveness Analysis in Class 3A Hospital Setting

The average cost of the antimicrobial-coated PICC in a Class 3 hospital was CNY 62,817.79, while the average price of the standard PICC was CNY 102,861.57. According to the calculated ICER, compared to the standard PICC, the antimicrobial-coated PICC resulted in an average cost saving of CNY 4,004,378.00 per QALY in a Class 3A hospital setting (Table 3).

In the standard group, the cost of fever of unknown origin was CNY 444,819.29, and that of CLABSI was CNY 444,404.50; in the antimicrobial-coated PICC group, the cost of unknown fever was CNY 314,742.50 and that of CLABSI was CNY 314,204.50. Thus, antimicrobial-coated PICC appears to be a more cost-effective solution.

#### 3.3.2. Cost-Effectiveness Analysis in Community Hospital Setting

During the remission phase of treatment, patients could transition to community hospitals for catheter maintenance, incurring lower care costs than in Class 3A hospitals. The average patient cost was CNY 61,235.43 for antimicrobial-coated PICCs and CNY 100,561.69 for standard PICCs. This resulted in a cost saving of CNY 3,932,626.00 per QALY.

**Figure 2:**
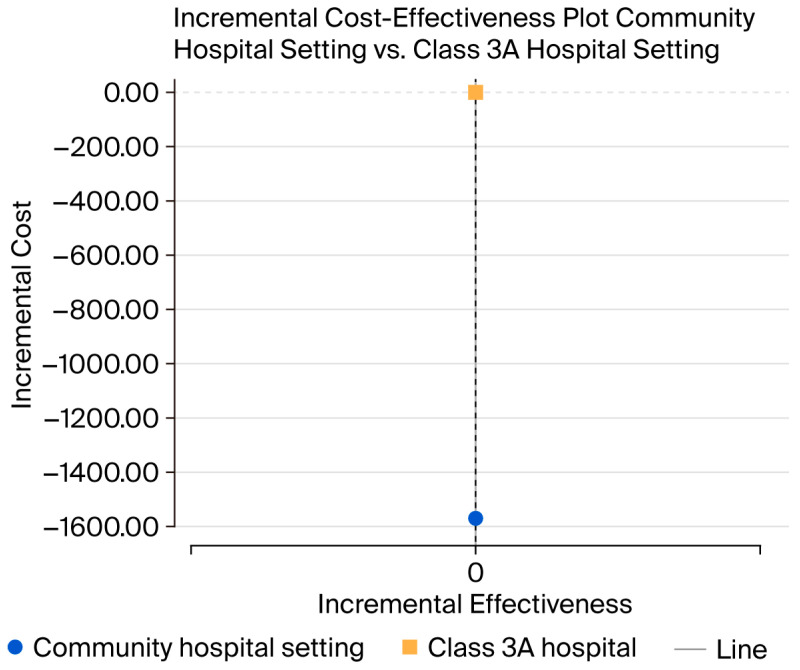
The incremental cost-effectiveness plot of Class 3A hospital setting vs. community hospital.

Although no significant difference in clinical outcomes was observed between the two care settings, community hospitals demonstrated clear cost savings (Figure 2). Specifically, the incremental cost in the community hospital was approximately CNY 1600 lower per patient. These findings highlight the economic advantage of managing long-term PICC care in community settings and support the potential for improved resource efficiency by decentralizing certain aspects of oncology care. Overall, antimicrobial-coated PICCs represent a more cost-effective solution for both hospital and community settings.

## 4. Discussion

Although PICC and CVC care has improved over the past two decades, catheter-related bloodstream infections (CLABSIs) and unexplained fevers still pose challenges [33,34]. Ongoing research is essential to advance catheter technology and optimize patient care strategies to reduce complications and healthcare costs. In this study, all eligible patients who received PICC insertions were included, though catheter type was not assigned randomly, which may have introduced selection bias. However, the indications for catheter placement and underlying disease status were similar across groups, supporting comparability. A decision tree model was used to assess cost-effectiveness, rather than a formal Delphi process. To ensure model reliability, we conducted both one-way and probabilistic sensitivity analyses, which demonstrated consistent results across a wide range of assumptions, affirming the robustness of the model.

Long-term indwelling catheters are generally considered safe, comfortable, and easy to manage. While implanted ports are widely used in Western countries, their adoption in China is limited due to high costs and the invasive nature of the procedure. This study evaluated the cost-effectiveness of two types of PICCs—standard and antimicrobial-coated—across both Class 3A hospitals and community hospital settings in China. The primary difference between these settings lies in the level of catheter maintenance care provided. If community hospitals in China expand their services—similar to those in the U.S.—additional cost savings could be realized.

Antimicrobial-coated PICCs were associated with lower CLABSI rates compared to standard PICCs, though differences in other clinical outcomes were not significant. However, the reduction in complication-related costs was substantial in both healthcare settings. These real-world findings highlight both the clinical and economic value of antimicrobial-coated PICCs, as well as the practical challenges of implementation across diverse care environments.

### 4.1. Implications for Practice and Policy

As new technologies emerge, clinicians must balance long-term benefits against initial costs. Antimicrobial-coated PICCs showed better performance in terms of complication control and safety. Policymakers should consider incorporating cost-effective PICCs into standard healthcare practices and coverage policies to enhance patient satisfaction and optimize resource utilization. Additionally, greater emphasis should be placed on community healthcare settings by enhancing training programs for healthcare providers and expanding services for long-term care patients. Such improvements could effectively address healthcare resource allocation imbalances and enhance care accessibility at the community level.

### 4.2. Future Research Directions

Given the extensive use of PICCs, future research should include multicenter studies to validate findings across different healthcare environments. Specific research priorities include the development of clear, innovative health economic evaluation frameworks tailored to supportive medical devices, and evaluation methodologies that consider variations in healthcare settings to allow broader analytical scopes for assessing the value of novel catheter technologies. Additionally, it is important to explore clinical scenarios where statistically non-significant outcomes may still represent meaningful economic or practical value in real-world practice.

Antimicrobial-coated PICCs demonstrated cost-effectiveness in this study; the findings suggest they are economically justified for high-risk patient groups from a societal perspective. These results underscore the need for policymakers to refine evaluation methods for novel medical devices, especially those that reduce complication rates. Lower complication rates can lead to significant reductions in healthcare costs and improvements in care quality across various healthcare settings.

While this study provides insights into China’s evolving healthcare system, future studies incorporating multi-country comparisons—particularly in settings with different reimbursement systems and care delivery models, such as Australia or the United States—are recommended to enhance external validity.

## 5. Limitation of the Study

This study possesses several strengths, including extensive data collection, application of a rigorous decision-analytical model, and validation via a Delphi panel. However, certain limitations must be acknowledged:

1. Use of QALYs: The quality-adjusted life years (QALYs) applied in this study were derived from previous hematology-related research measuring general treatment impacts on patient quality of life. However, this approach did not specifically isolate catheter-associated quality-of-life variations, nor did it incorporate patient-reported outcomes (PROs) related to catheter function, discomfort, or complication burden. As a result, potential differences in patient experience between catheter types may not be fully captured. Future research should include direct collection of catheter-specific quality-of-life data, catheter-specific PROs and utility weights to evaluate catheter impacts more accurately.
2. Economic evaluation methods: Traditional economic evaluation methodologies may not fully capture the value of interventions primarily to enhance patient quality of life without significantly altering disease progression. Innovative economic evaluation theories and analytical tools are necessary to assess the true economic value of supportive medical technologies adequately.

## Figures and Tables

**Table 1:** Clinical variables of patients in the two PICC groups.

Observation Item		Antimicrobial-Coated PICC (*n* = 113) *n* (%)	Standard PICC (*n* = 111) *n* (%)	Total (*n* = 224) *n* (%)	*p*-Value ^a^
**Gender**					0.513
	Male	71 (62.83%)	65 (58.56%)	136 (60.71%)	
	Female	42 (37.17%)	46 (41.44%)	88 (39.29%)	
**Age ^b^**		41.36 (12.98)	43.34 (14.38)	42.34 (13.70)	0.280
**BMI ^b^**		22.97 (3.21)	24.03 (3.40)	23.50 (3.34)	0.020
**APTT ^b^**		31.13 (3.77)	30.58 (3.41)	30.86 (3.60)	0.251
**INR ^b^**		1.05 (0.10)	1.06 (0.13)	1.06 (0.11)	0.852
**Catheter information**					
**The 1st puncture success**					0.304
	1st puncture success	112 (99.12%)	108 (97.30%)	220 (98.05%)	
	No 1st puncture success	1 (0.88%)	3 (2.70%)	4 (1.95%)	
**Puncture arm**					0.605
	Left arm	42 (37.17%)	45 (40.54%)	87 (38.84%)	
	Right arm	71 (62.83%)	66 (59.46%)	137 (61.16%)	
**Catheter size**					0.834
	4.0–4.5 French	55 (48.67%)	52 (47.27%)	107 (47.98%)	
	5.0–5.5 French	58 (51.33%)	58 (52.73%)	116 (52.02%)	
**Catheter lumen**					0.894
	Single lumen	56 (49.56%)	56 (50.45%)	112 (50.00%)	
	Double lumen	57 (50.44%)	55 (49.55%)	112 (50.00%)	
**Indwell Period (Days) ^b^**		62.81 (±27.98)	69.04 (±26.65)	65.89 (±27.44)	0.089
**Catheter-related complications**					
**CLABSI**					0.076
	Non-CLABSI	112 (100.00%)	105 (97.22%)	217 (98.65%)	
	CLABSI	0 (0.00%)	3 (2.65%)	3 (1.32%)	
**Unknown Fever**					0.449
	No	95 (84.82%)	85 (80.95%)	180 (82.95%)	
	Yes	17 (15.18%)	20 (19.05%)	32 (17.05%)	
**Other local complications**					0.449
	Non complications	95 (84.82%)	85 (80.95%)	180 (82.95%)	
	Catheter-related complications	17 (15.18%)	20 (19.05%)	37 (17.05%)	

Antibacterial-coated PICCs were used in the study group and Standard PICCs were used in the control group. ^a^ Pearson’s chi-square test for heterogeneity. ^b^ Values are presented as mean and SD compared with a *t*-test. SD: Standard deviation. Statistical significance at *p* < 0.05.

**Table 2:** Cost variables used in cost-effectiveness model.

Model Input		Base-Case Value	Source
**Cost**			
	Price of Standard PICC (CNY/piece)	2100.00	Hospital data
	Estimated Price of Antimicrobial PICC (CNY/piece)	2300.00	Estimated data
	Catheter maintenance (CNY/per patient) in 90 days		
	In Class 3A hospital	2100.11	Hospital data
	In community hospital	1200	Calculated
	Catheter insertion/replacement (CNY/per patient)	364.25	Hospital data
	CLABSI Diagnosis (CNY/per time)	1332.77	Hospital data
	CLABSI Treatment (CNY/per time)	87,147.08	Hospital data
	Hospitalization per day in Beijing Class 3A hospital (CNY/per bed per day)	200.00	Hospital data
	QALY of pre-treatment	0.65	Liang Y, Wang H et al., 2018 [28]
	QALY of treatment	0.90	Liang Y, Wang H et al., 2018 [28]
**Length of stay, day**			
	Patient with CLABSI	20.6	Hospital data
	Patient without CLABSI	11.2	Hospital data

**Table 3:** Cost-effectiveness analysis results of antimicrobial-coated PICCs vs. standard PICCs in Class 3A hospital and community hospital settings.

Expenses Based on Different Situations (In 90 Days)	Antimicrobial-Coated PICCs (*n* = 112)		Standard PICCs (*n* = 108)		ICER	*p*-Value
	Costs	Probability	Costs	Probability	(Antimicrobial vs. Standard)	
**Total expense in Class 3A Hospital**	62,817.79	100.00%	102,861.57	100.00%	(4,004,378.00)	0.001
Puncture unsuccess	17,328.50	0.98%	18,928.50	2.91%		
CLABSI	314,204.50	0.00%	444,404.50	2.91%		
Unknown fever	314,742.50	16.67%	444,819.29	18.45%		
Other local catheter-related complications	30,964.25	13.72%	14,374.87	2.91%		
Complications-free	8664.25	68.63%	9464.25	72.81%		
**Total expense in Community Hospital**	61,235.43	100.00%	100,561.69	100.00%	(3,932,626.00)	

Statistically significant at *p* < 0.05.

## Data Availability

All data are provided within the manuscript.

## Abbreviations

The following abbreviations are used in this manuscript:

CLABSI	Central line-associated bloodstream infection
QALYs	Quality-adjusted life years
ICER	Three letter acronym
CEA	Cost-Effectiveness Analysis
PICC	Peripherally inserted central catheter
